# Does perfusion computed tomography correlate to pathology in colorectal liver metastases?

**DOI:** 10.1371/journal.pone.0245764

**Published:** 2021-01-26

**Authors:** M. J. van Amerongen, A. M. Vos, W. van der Woude, I. D. Nagtegaal, J. H. W. de Wilt, J. J. Fütterer, J. J. Hermans

**Affiliations:** 1 Department of Radiology and Nuclear Medicine, Radboud University Nijmegen Medical Centre, Nijmegen, The Netherlands; 2 Department of Pathology, Radboud University Nijmegen Medical Centre, Nijmegen, The Netherlands; 3 Department of Surgery, Radboud University Nijmegen Medical Centre, Nijmegen, The Netherlands; Medical University of Vienna, AUSTRIA

## Abstract

**Introduction:**

Targeted therapy against tumor angiogenesis is widely used in clinical practice for patients with colorectal liver metastases (CRLM). Possible predictive biomarkers for tumor angiogenesis, such as, microvessel density (MVD), hypoxia and cell proliferation, can be determined using immunohistochemical staining. However, patients ineligible for surgical treatment need to undergo invasive diagnostic interventions in order to determine these biomarkers. CT perfusion (CTP) is an emerging functional imaging technique, which can non-invasively determine vascular properties of solid tumors. The purpose of this study was to evaluate CTP with histological biomarkers in CRLM.

**Material and methods:**

Patients with CRLM underwent CTP one day before liver surgery. CTP analysis was performed on the entire volume of the largest metastases in each patient. Dual-input maximum slope analysis was used and data concerning arterial flow (AF), portal flow (PF) and perfusion index (PI) were recorded. Immunohistochemical staining with CD34, M75/CA-IX and MIB-1 was performed on the rim in the midsection of the tumor to determine respectively MVD, hypoxia and cell proliferation.

**Results:**

Twenty CRLM in 20 patients were studied. Mean size of the largest CRLM was 37 mm (95% CI 21–54 mm). Mean AF and PF were respectively 64 ml/min/100ml (95% CI 48–79) and 30 ml/min/100ml (95% CI 22–38). Mean PI was 68% (95% CI 62–73). No significant correlation was found between tumor growth patterns and CTP (p = 0.95). MVD did not significantly correlate to AF (r = 0.05; p = 0.84), PF (r = 0.17; p = 0.47) and PI (r = -0.12; p = 0.63). Cell proliferation also did not significantly correlate to AF (r = 0.07; p = 0.78), PF (r = -0.01; p = 0.95) and PI (r = 0.15; p = 0.52). Hypoxia did not significantly correlate to AF (r = -0.05; p = 0.83), however, significantly to PF (r = 0.51; p = 0.02) and a trend to negative correlation with PF (r = -0.43; p = 0.06). However, after controlling the false discovery rate, no significant correlation between CTP and used immunohistochemical biomarkers was found.

**Conclusion:**

In conclusion, this feasibility study found a trend to negative correlation between PI and hypoxia, CTP might therefore possibly evaluate this prognostic marker in CRLM non-invasively. However, CTP is not an appropriate technique for the assessment of microvessels or cell proliferation in CRLM.

## Introduction

Liver metastases occur frequently in patients with colorectal cancer [1–3]. Growth and spread of these metastases are greatly dependent on their ability to induce and maintain vascular perfusion and, for this reason, vascular perfusion is associated with a poor outcome in colorectal cancer [4]. Targeted therapy against tumor angiogenesis has been developed in order to improve survival in patients with colorectal cancer and is widely applied in clinical practice [5]. Assessment of tumor behavior could therefore also play an important role in the clinical management as it could potentially provide an estimation of patient’s prognosis [6]. However, there is still a lack in studies on possible predictive biomarkers for adequate patient selection [7]. Possible predictive biomarkers, for example, microvessel density (MVD), tumor hypoxia and cell proliferation have been investigated, of which MVD is one of the most used techniques in order to quantify tumor angiogenesis, as it is believed to summarize the effect of all different angiogenic regulators [8]. MVD, tumor hypoxia and cell proliferation can be assessed using different immunohistochemical stainings on resection specimens [9–11]. However, in patients with CRLM that are not eligible for surgical treatment, additional invasive diagnostic interventions are necessary to determine these possible predictive biomarkers.

An emerging functional imaging technique is CT perfusion (CTP). This CT technique measures the temporal changes in tissue and vascular enhancement after intravenous administration of contrast material, after which different mathematical models can be used, compartmental or deconvolution, in order to quantify vascular parameters [12]. By acquiring these parameters of studied tissue, CTP is able to provide additional information regarding tumor vascularity beside the tumor morphology acquired in a normal/ standard CT. It remains unclear whether these CTP parameters can reliably be used in the management of patients with CRLM. For this purpose, CTP parameters can be compared to known predictive biomarkers, such as MVD, hypoxia and cell proliferation and therefore possibly omit future biopsy in these patients. However, in the current literature, no consensus exists concerning the correlation between MVD and CTP. Goh et al. [13] reported a positive correlation between CTP and MVD in primary colorectal cancer (CRC), however multiple other studies did not find a correlation between CTP and MVD in these tumors [14–16]. Also, these studies used a deconvolution analysis and were performed on primary CRC and not CRLM. To our knowledge, the correlation between tumor hypoxia, cell proliferation and CTP parameters in CRLM has not been studied in the literature. Because of the contradictory correlation between MVD and CTP in primary CRC and the absence of studies comparing compartmental CTP to pathological markers in CRLM, this study aims to evaluate compartmental CTP parameters with pathological biomarkers in CRLM.

## Materials and methods

### Patients

Consecutive patients with CRLM, who were scheduled for metastasectomy, were included for this study. Patients were only included when 18 years or older and with a Karnofsky score of 70. Patients were excluded if they presented with contra-indications for contrast enhanced CT, e.g., when they were known with iodine allergy, pregnancy or renal disease (Glomerular Filtration Rate < 60 ml/min/1.73m^2^) or received neoadjuvant chemotherapy less than 6 weeks before operation. This study was approved by our Institutional Review Board (CMO region Arnhem-Nijmegen; NL number 37784.091.11) and all patients gave written informed consent.

### CT imaging

All patients received CTP of the liver one day before initial planned liver surgery with the use of a 320-slice CT scanner (Aquilion ONE, Canon Medical Systems). Dynamic imaging was only performed within the 16 cm detector range of the scanner. A non-contrast helical CT was performed of all patients in order to ensure the coverage of the metastases within the 16 cm scan range for further dynamic imaging. A scanning protocol was chosen combined with a diagnostic venous phase liver CT and perfusion imaging in one session, e.g., diagnostic images were acquired between the dynamic time points. The CT had an X-Ray tube voltage of 100 kV. The milliampere-seconds was set at 50 mAs or Automatic Exposure Control for respectively the dynamic and diagnostic imaging. Rotation time was 0.5 seconds with a pitch of 0. Patients received 150 ml of iodine contrast at a concentration of 300 mg/ml at an injection rate of 5 ml/s (Iomeron® Bracco UK Ltd.) followed by a saline flush (Mallinckrodt /Covidien OptiVantage Power Injector). Imaging was performed during 110 seconds with 22 time points starting 7 seconds after contrast injection. Median DLP was 775 mGy.cm (range 347–1367).

### Image analysis

CT images were transferred to a workstation, i.e., Vitrea® Advanced Visualization Software (V7, Vital Images Inc. (a Canon group company), Minnetonka, MN, USA). This software automatically registered the liver and corrected for respiratory motion changes. Afterwards, the perfusion maps were calculated by a radiologist using a compartmental algorithm, i.e., a dual input maximum slope model, after initial non-rigid registration by the workstation. A circular region of interest (ROI) was placed inside the aorta, the portal vein, the spleen and healthy liver tissue in order to compute the dual-input maximum slope for each patient. After computing the model, the workstation provided mean arterial flow (AF), portal flow (PF) and the perfusion index (PI), i.e., AF/(AF+PF)*100%, within specified regions. The metastases were identified on CE-CT. Perfusion values of both the tumor and normal liver tissue were measured by placing an ROI in the respective tissue at three different locations in both the axial and coronal plane on the CE-CT images. Automatic values concerning AF, PF and PI at these locations were calculated by the software. Mean values of AF, PF, PI and total perfusion (AF+PF) of these six measurements were calculated for both the tumor and normal tissue. The PI of the tumor was further categorized in four categories (1: 0–25%, 2: 25–50%, 3: 50–75% and 4: 75–100%).

### Pathological analysis

**Fig 1:**
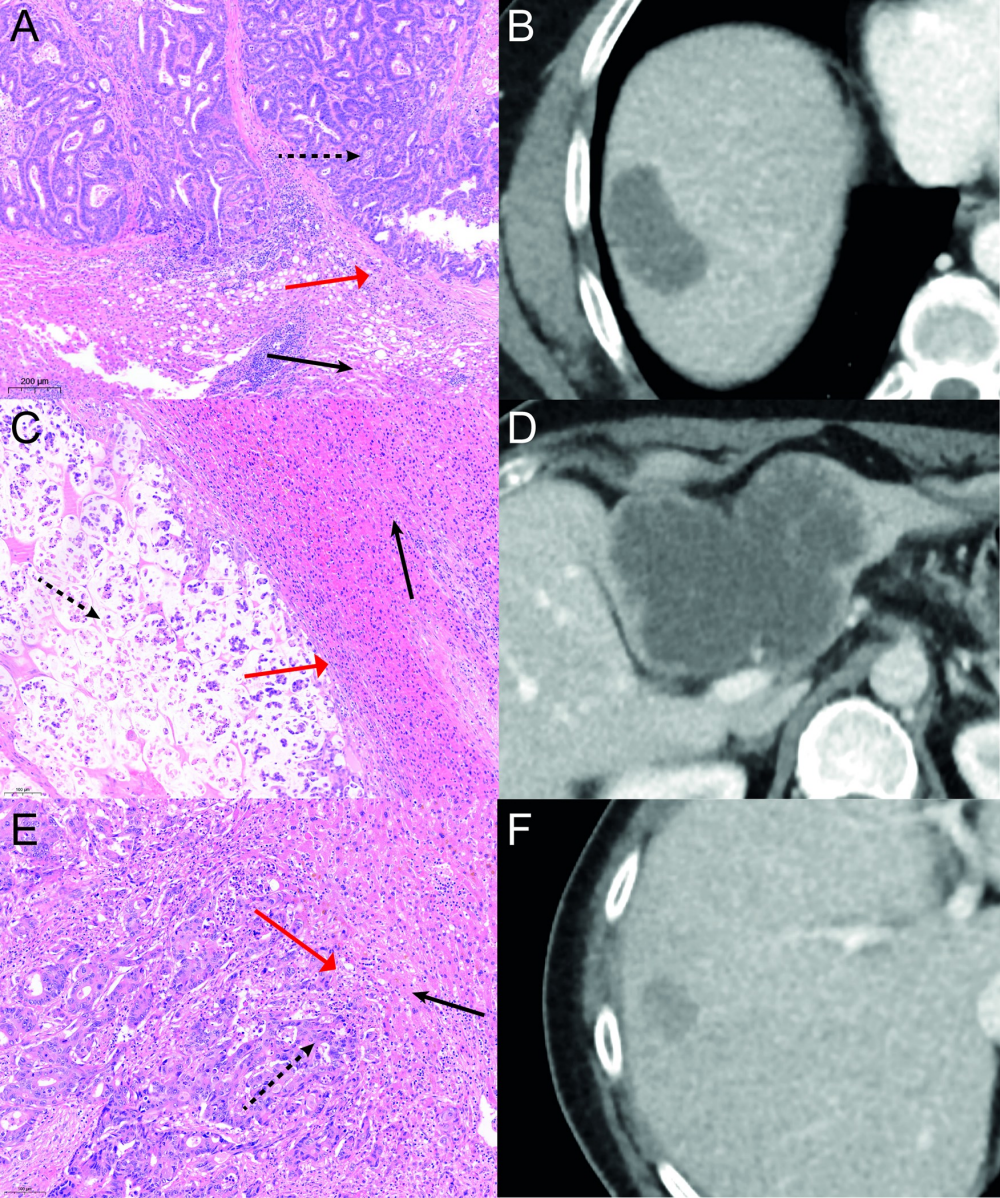
Growth patterns. Growth patterns of CRLM in different patients. A-B: HE staining showing a desmoplastic growth pattern with corresponding a CT in venous phase. On the pathology images fibrous tissue (red arrow) is visible between tumor (dashed arrow) and normal liver tissue (black arrow). C-D: A pushing growth pattern is visible on the HE staining with its corresponding venous phase CT. Between tumor (dashed arrow) and normal tissue (black arrow) compression is visible of normal cells (red arrow). E-F: HE staining with corresponding CT in venous phase showing a replacement growth pattern. No clear border between tumor (dashed arrow) and healthy tissue (black arrow) is visible. Tumor cells can be seen between normal tissue (red arrow).

After surgical resection the specimen was fixed with 10% formaldehyde and embedded in paraffin. The tumor was analyzed by the pathology department for histologic grade (high, intermediate and low grade) and growth patterns (desmoplastic, pushing or replacement), see Fig 1.

**Fig 2:**
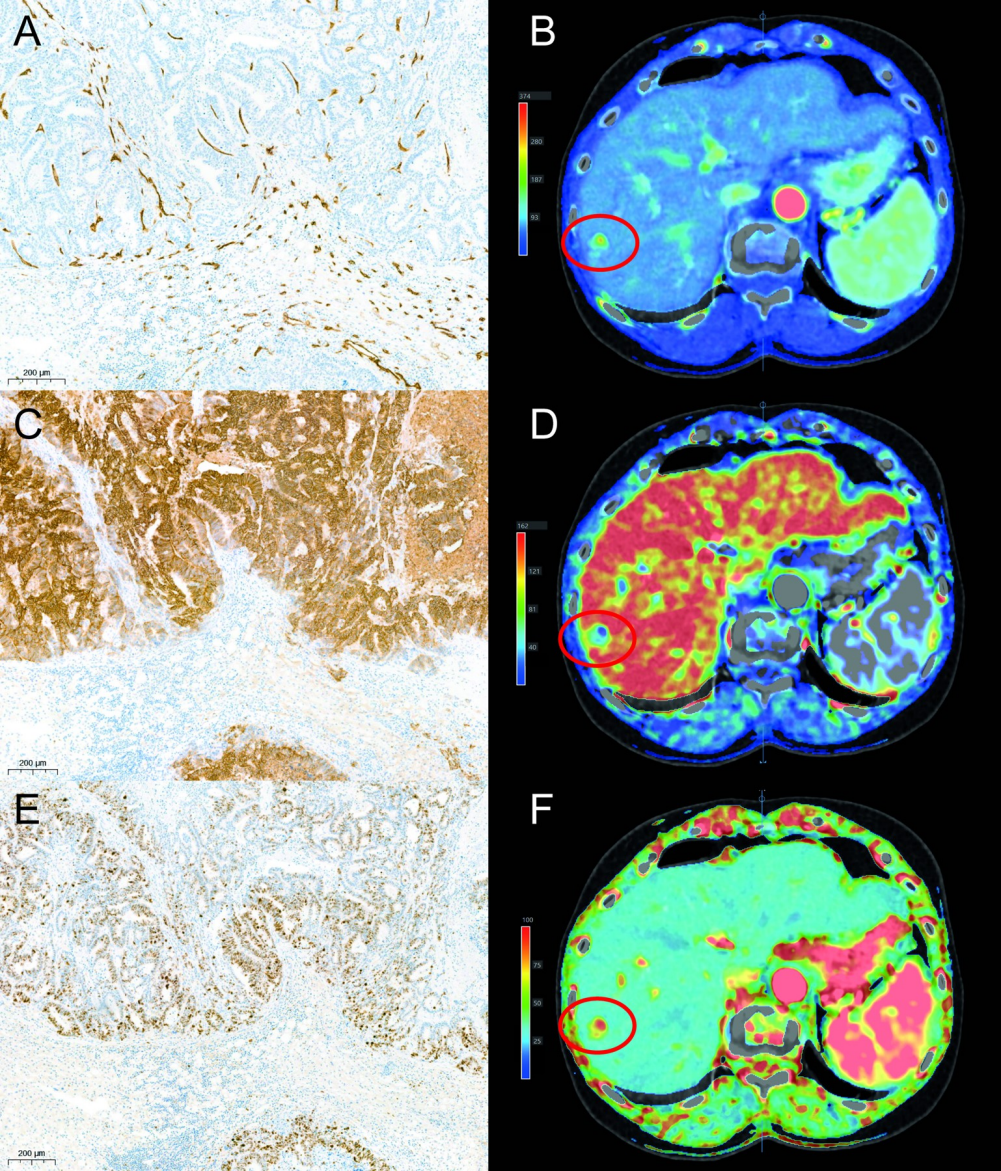
Immunohistochemical stainings. Immunohisochemical stainings in one patient. Marker CD34 showing microvessels in brown (A). Marker M75 showing the expression of CA-IX on cells which is associated with hypoxia. Cells showing CA-IX on their membranes are depicted in brown (C). Staining with the marker MIB-1 which shows cells in mitosis in brown (E). Example of perfusion values acquired with the arterial flow (B), portal flow (D) and perfusion index (F). The tumor is highlighted with a red circle.

For all cases formalin-fixed paraffin-embedded (FFPE) tumor tissue samples were available. These samples were cut into 4μm thick sections for immunohistochemical slides. The slides were processed on an automated immunostainer (Autostainer 360, Thermo Scientific, Waltham, Massachusetts, USA; or Tissue-Tek Genie, Sakura Finetek USA Inc, Torrance, California, USA), in which the tissue sections were deparaffinized in xylene and then rehydrated with ethanol. Antigen retrieval was done by boiling in ethylenediaminetetraacetic acid (EDTA) buffer (pH 9.0) at 97°C. Markers CD34 (Tissue-Tek Genie®; Sakura Finetek, USA), M75/ CA-IX (Department of Urology, Radboudumc, Nijmegen, The Netherlands; clone M75; dilution 1:4000) and MIB-1 (DakoCytomation, Glostrup, Denmark; clone Ki-67; dilution 1:25) were used to quantify respectively MVD, hypoxia and cell proliferation of sections of the tumor at the rim of the midtumor region. Positive and negative controls were used in each staining. The different immunohistochemical stainings are visualized in Fig 2.

For the quantitative analysis of CD34, hot spot analysis was used for microvessel counting [17]. Low-power magnification (x 40) was used to examine the slides initially and to identify areas with a high density of microvessels. Three area’s with the highest vascularization were selected. Using a high-power magnification (x 200) field of each of these areas, the microvessels were counted inside a 1 x 1 mm square, at 3 different location inside the area. The average of these 9 different counts was recorded for each case and represented the MVD.

The fraction of highlighted tumor cells after immunohistochemical staining with M75/ CA-IX and MIB-1 were estimated using a high-power magnification (x200) field, representing respectively hypoxia and cell proliferation. The immunostaining of M75/ CA-IX was additionally semiquantitatively scored: 0 = no staining, 1 = weak staining, 2 = moderate staining and 3 = strong staining [18].

### Statistical analysis

Continuous variables are expressed as means ± Interquartile range. The Mann-Whitney U test was used to calculate the differences in perfusion values between normal tissue and CRLM. Q-Q plots were generated concerning perfusion parameters and immunohistochemical staining in order to test for normal distribution. The correlation between the continuous variables of MVD, percentage of hypoxia, cell proliferation and the perfusion parameters, i.e., AF, PF and PI, was analyzed using Pearson’s correlation analysis. The Benjamini & Hochberg method was used on the p-values after the correlation analysis for controlling the false discovery rate. The PI parameter was categorized in 4 groups (group 1: 0–25%, group 2: 25–50%, group 3: 50–75% and group 4: 75–100%), after which the Chi squared test was used for the evaluation of the histopathological differentiation grade, growth patterns and intensity of M75/ CA-IX staining. Analysis was performed using the Statistical Package for the Social Sciences (SPSS, version 20; SPSS, Chicago, IL, USA). A p-value smaller than 0.05 was considered significant.

## Results

### Patients

Twenty patients were included in this prospective study, of which 15 men and 5 women. Twelve patients presented with a primary rectal cancer and 8 patients with colon cancer. Mean age was 65 years (95% CI 61–70 years). Patients presented with 37 liver metastases with an average diameter of 37 mm (95% CI 21–54 mm). For each patient, the largest tumor was evaluated with CT perfusion and immunohistochemical stainings, meaning 20 tumors were analyzed. Patient demographics are summarized in Table 1.

**Table 1:** Patient demographics.

		Included patients (n = 20)
*Age (mean)*		65 years (95% CI 61–70 years)
*Gender (M:F)*		15:5
*ASA*	Class 1	1 (10%)
	Class 2	18 (90%)
	Class 3	1 (10%)
*BMI*		28 kg/m^2^ (95% CI 26–30)
*Location primary (colon: rectum)*		8:12
*Number CRLM*		37 metastases
*Single metastasis: multiple metastases*		12:8
*Size largest CRLM*		37 mm (95% CI 21–54 mm)
*Neo-adjuvant chemotherapy*		4 (20%)
*Radicality of surgical resection (R0%)*		19 (95%)

### CT perfusion

Normal liver tissue showed a mean AF and PF of respectively 71 ml/min/100 ml (95% CI 65–77) and 134 ml/min/100 ml (95% CI 115–154). The mean PI of normal liver tissue was 36% (95% CI 31–40) and mean total perfusion (AF+PF) was 205 ml/min/100 ml (95% CI 188–223). The difference between the AF in tumor and healthy tissue was non-significant (p = 0.05). However, the lower PF, higher PI and lower total perfusion of CRLM were statistically significant compared to normal liver tissue (all with p = 0.001). Differences in perfusion values between normal tissue an tumor are depicted in S1 Fig.

The colorectal liver metastases showed a mean HU value of 81 (95% CI 69–94). Mean AF and PF of the metastases were respectively 64 ml/min/100 ml (95% CI 48–79) and 30 ml/min/100 ml (95% CI 22–38). Mean PI was 68% (95% CI 62–73). Three patients were included in PI category 2, twelve patients in category 3 and five patients in category 4. No patients were included in category 1. Mean total perfusion (AF+PF) was 94 ml/min/100 ml (95% CI 74–113) in studied CRLM.

### Immunohistochemical staining and pathological evaluation

Nineteen tumors were non-mucinous liver metastases and one tumor was mucinous. Thirteen patients presented with well differentiated metastases, five patients with moderate differentiation and two metastases showed poor differentiation. Twelve metastases showed a desmoplastic growth pattern, 3 metastases a pushing growth pattern and a replacement growth pattern was shown in 5 patients. Mean MVD was 65 (CD34; 95% CI 56–73). Mean fraction of the hypoxia marker M75/ CA-IX and the proliferation marker MIB-1 was respectively 29% (95% CI 17–40) and 56% (95% CI 44–68). Four patients showed weak staining (score 1) of the hypoxia marker M75/ CA-IX, eleven patients moderate staining (score 2) and four patients strong staining (score 3). One patient showed no staining after application of M75/ CA-IX (score 0).

### Correlation between CT perfusion and pathology in CRLM

**Fig 3:**
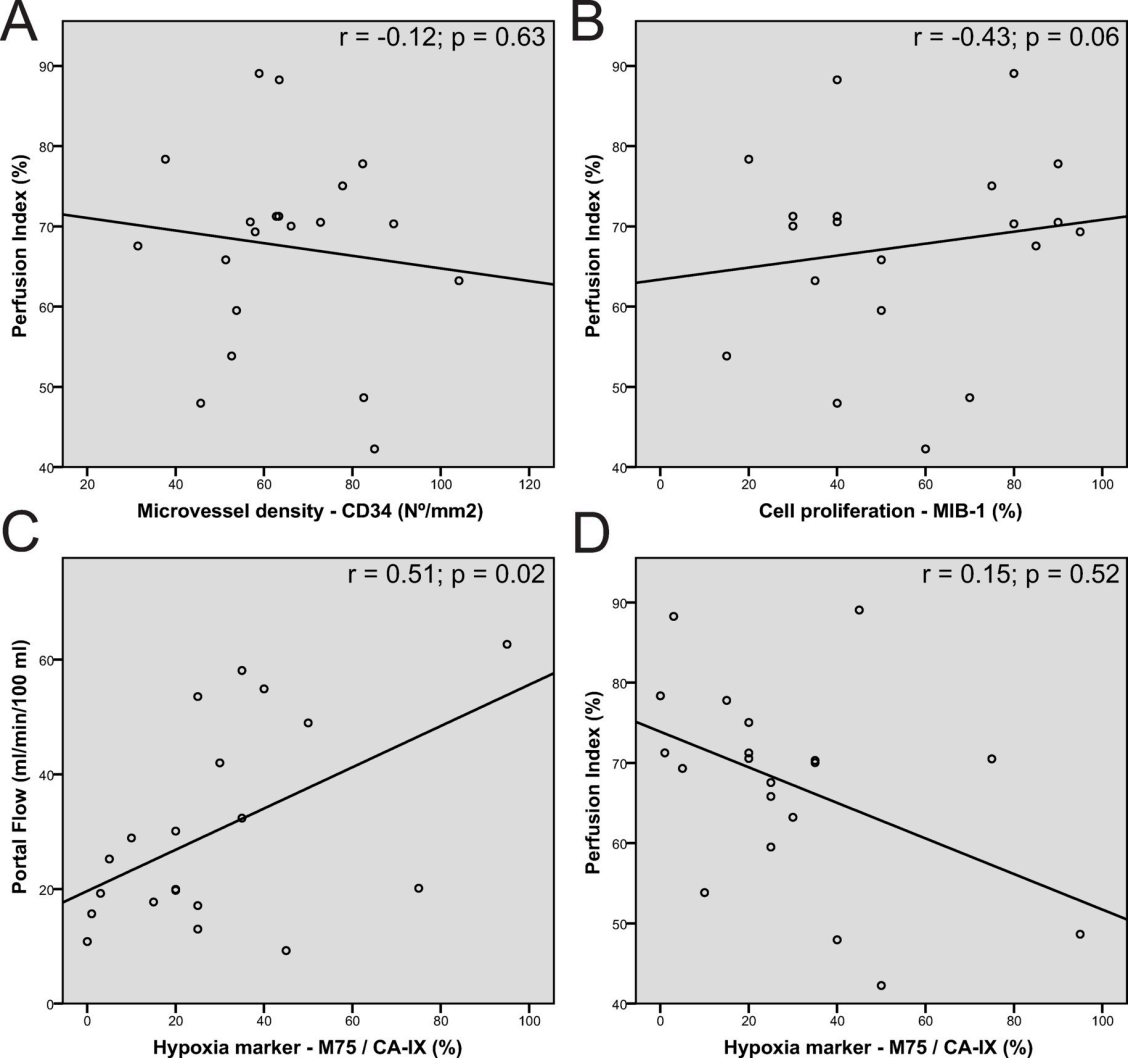
Scatter plots between perfusion parameters and pathology markers. Scatter plots between Perfusion index and MVD (A); Perfusion index and cell proliferation (B) and Portal flow/ Perfusion index and hypoxia (CD). The different plots show a large spread of different points with a low magnitude in the regression line. However, a steep positive regression line was seen between portal flow and hypoxia (C) and between perfusion index and hypoxia (D), corresponding to the respectively significant positive correlation and trend to negative correlation between these parameters.

Using Pearson’s correlation, the PI did not show a significant correlation with MVD (r = -0.12; p = 0.63) and cell proliferation (r = 0.15; p = 0.52). A trend to statistical significance was shown between PI and hypoxia (r = -0.43; p = 0.06). The AF did not show significant correlation with the immunohistochemical staining (MVD r = 0.05; p = 0.84, hypoxia r = -0.05; p = 0.83 and cell proliferation r = 0.07; p = 0.78). PF showed a significant correlation with the hypoxia (r = 0.51, p = 0.02), however, no significant correlation with MVD (r = 0.17, p = 0.47) and cell proliferation (r = -0.01; p = 0.95). After controlling the false discovery rate, no significant correlation between CTP and used immunohistochemical stainings was found. Correlation values are depicted in Fig 3.

The PI categories did not significantly differ in differentiation grade or growth pattern (respectively p = 0.69 and p = p = 0.95). Intensity of M75/ CA-IX staining was not statistically different between the PI categories (p = 0.21). Comparison between CT perfusion and these pathological outcomes are shown in Table 2.

**Table 2:** Comparison perfusion index to pathology.

		PI categories				p-value
		1 (n = 0)	2 (n = 3)	3 (n = 12)	4 (n = 5)	
*Differentiation*	Well	0 (0%)	2 (67%)	8 (67%)	3 (60%)	0.69
	Intermediate	0 (0%)	1 (33%)	2 (17%)	2 (40%)	
	Low	0 (0%)	0 (0%)	2 (17%)	0 (0%)	
*Growth pattern*	Desmoplastic	0 (0%)	2 (67%)	7 (58%)	3 (60%)	0.95
	Pushing	0 (0%)	0 (0%)	2 (17%)	1 (20%)	
	Replacement	0 (0%)	1 (33%)	3 (25%)	1 (20%)	
*Intensity M75/ CA-IX staining*	Score 0	0 (0%)	0 (0%)	0 (0%)	1 (20%)	0.21
	Score 1	0 (0%)	0 (0%)	3 (25%)	1 (20%)	
	Score 2	0 (0%)	1 (33%)	7 (58%)	3 (60%)	
	Score 3	0 (0%)	2 (67%)	2 (17%)	0 (0%)	

PI = perfusion index; M75 / CA-IX = hypoxia marker.

## Discussion

In the present study, the correlation between compartmental CTP and histologic parameters in 20 patients with CRLM was analyzed. The dual input maximum slope CTP did not significantly correlate with MVD or cell proliferation in CRLM. However, a trend to negative correlation between the PI and the immunohistochemical marker for hypoxia was observed.

Dynamic contrast-enhanced (DCE) imaging techniques have been used for the evaluation of the microvasculature and for determining the effect of anti-angiogenic treatments [19–21]. In both CTP and DCE-MRI the principle is similar. Depending on the size of the agent, the distribution, which depends on agent size, into the interstitial space is observed. After mathematical modeling the perfusion parameters are then acquired. However, differences between the pharmacokinetic properties between CT and MRI contrast agents may affect the diffusion behaviors during DCE imaging [22]. Also, signal intensity of DCE-MRI needs special conversion in order to estimate the concentration of contrast agent and is highly dependent on acquisition parameters and different MRI scanners, making the technique less reproducible between different hospitals [19]. Because CT is generally more easily accessible compared to MRI, different studies have evaluated CTP compared to DCE-MRI for the evaluation of tissue microvasculature [23, 24]. To our knowledge, no studies were performed evaluating the correlation between CTP and MVD in CRLM. However, the correlation between CTP and MVD was evaluated in primary CRC. Of the four different studies in current literature, only one [13] found a positive correlation between CTP and MVD in primary CRC, suggesting that CTP is an appropriate technique for the assessment of vascularity in CRC. However, the remaining articles [14–16] did not find a correlation between these parameters in primary CRC. By using a dual-input maximum slope analysis, our study shows a lower portal flow and general perfusion in colorectal liver metastases compared to normal hepatic tissue, conform earlier studies [25, 26]. However, no significant correlation was demonstrated between researched markers and perfusion parameters, which is in line with the latter multiple studies regarding CTP in primary CRC. In the analysis a high MVD was expected to show a high PI, which was shown in our included patients, however, multiple patients also showed a high PI with a low number of microvessels, mainly due to the abundance of large vessels in the tumor, thereby increasing the perfusion values in the tumor. Probably for this reason, MVD did not show a significant correlation with the CTP parameters.

In our study, a trend to negative correlation was demonstrated between PI and hypoxia (r = -0.43; p = 0.06), which can also indirectly be used to assess tumor angiogenesis. No significant correlation was found between AF and hypoxia (r = -0.05; p = 0.83) and a significant positive correlation between PF and hypoxia (r = 0.51; p = 0.02) was observed. Perfusion in the liver is a collaboration between both the arterial and portal flow. A lower PF was associated with higher AF in evaluated patients. Because of the difference in correlation between AF and PF with hypoxia we additionally controlled the false discovery rate using the Benjamini & Hochberg method. Using this method, no statistical difference was shown between CTP and hypoxia. In current literature, in multiple MRI oriented studies, MRI was able to adequately assess the level of hypoxia in cholangiocarcinoma [27] and pancreatic [28] tumors. To our knowledge, however, no study evaluated CTP and hypoxia in CRLM. The lack of significant correlation between PI and the used hypoxia marker in the current study, could be explained by the number of included patients, possibly with a larger patient sample, this result could show significance. Therefore, this CTP parameter could potentially be used for the assessment of hypoxia. However, the lack of significant correlation between CTP and hypoxia could also partly be explained by the Warburg effect [29], i.e., the process of glycolysis in the presence of oxygen. Tumor cells often produce energy using aerobic glycolysis [30], causing increased production of lactate, CO_2_ and H^+^. Hypoxia marker M75/ CA-IX plays a crucial role in the pH regulation and is expressed on cell membranes during glycolysis in order to prevent acidosis [31]. This means that tumor cells can present M75/ CA-IX on their membranes despite adequate perfusion.

This study did not show any significant correlation between CTP and cell proliferation. In current literature, cell proliferation of different tumors is often evaluated in MRI studies. For example, in breast cancer it can radiologically be assessed by measuring the choline peak inside the tumor by using MR spectroscopy [32, 33]. Additionally, diffusion weighted imaging (DWI) with the accompanying apparent diffusion coefficient (ADC) can be used to measure restricted water diffusion and is inversely related to cell proliferation [32, 33]. However, CTP determines the blood flow in the capillary networks of examined tissue and is therefore more comparable to dynamic contrast enhanced MRI (DCE-MRI) [34]. No articles are currently available in literature evaluating the correlation between cell proliferation and CTP or DCE-MRI. Regarding the lack of correlation in this study and the absence of data in the literature, CTP is therefore deemed not suitable for evaluation of cell proliferation in CRLM.

The current study has some limitations, only a small number of patients could be analyzed, especially when comparing the different PI categories. Also, it was impossible to provide a precise correlation between CTP and the pathologic specimen. The CTP evaluated the entirety of the tumor, while only a smaller portion of the specimen was analyzed by the pathologist, meaning sampling bias could play a role. The analysis can be improved by assessing the whole-volume of the specimen, however, this can currently only be done manually and is therefore very labor intensive and not feasible. Possibly, in the future, automatic software is available for these assessments. Lastly, the current study only used dual-input maximum slope analysis as the computer, on which the analysis was performed, was not able to perform a deconvolution analysis for the liver.

In conclusion, this feasibility study found a trend to negative correlation between PI and hypoxia, CTP might therefore possibly evaluate this prognostic marker in CRLM non-invasively. However, CTP is not an appropriate technique for the assessment of microvessels or cell proliferation in CRLM.

## Supporting information

S1 Fig Perfusion differences between tumor and normal liver tissue. Box plots showing difference in arterial flow (A), portal flow (B), perfusion index (C) and the total perfusion (D) between tumor and normal hepatic tissue of studied patients. No significant difference was shown in arterial flow. Perfusion values of the tumor showed significantly lower portal flow, higher perfusion index and lower total perfusion compared to normal liver tissue.
(TIF)

S1 File (SAV)
