# Supplementary figures and images for: Does perfusion computed tomography correlate to pathology in colorectal liver metastases?
Source: PLoS One. 2021 Jan 26;16(1):e0245764. doi: 10.1371/journal.pone.0245764 (PMC7837475; doi:10.1371/journal.pone.0245764)

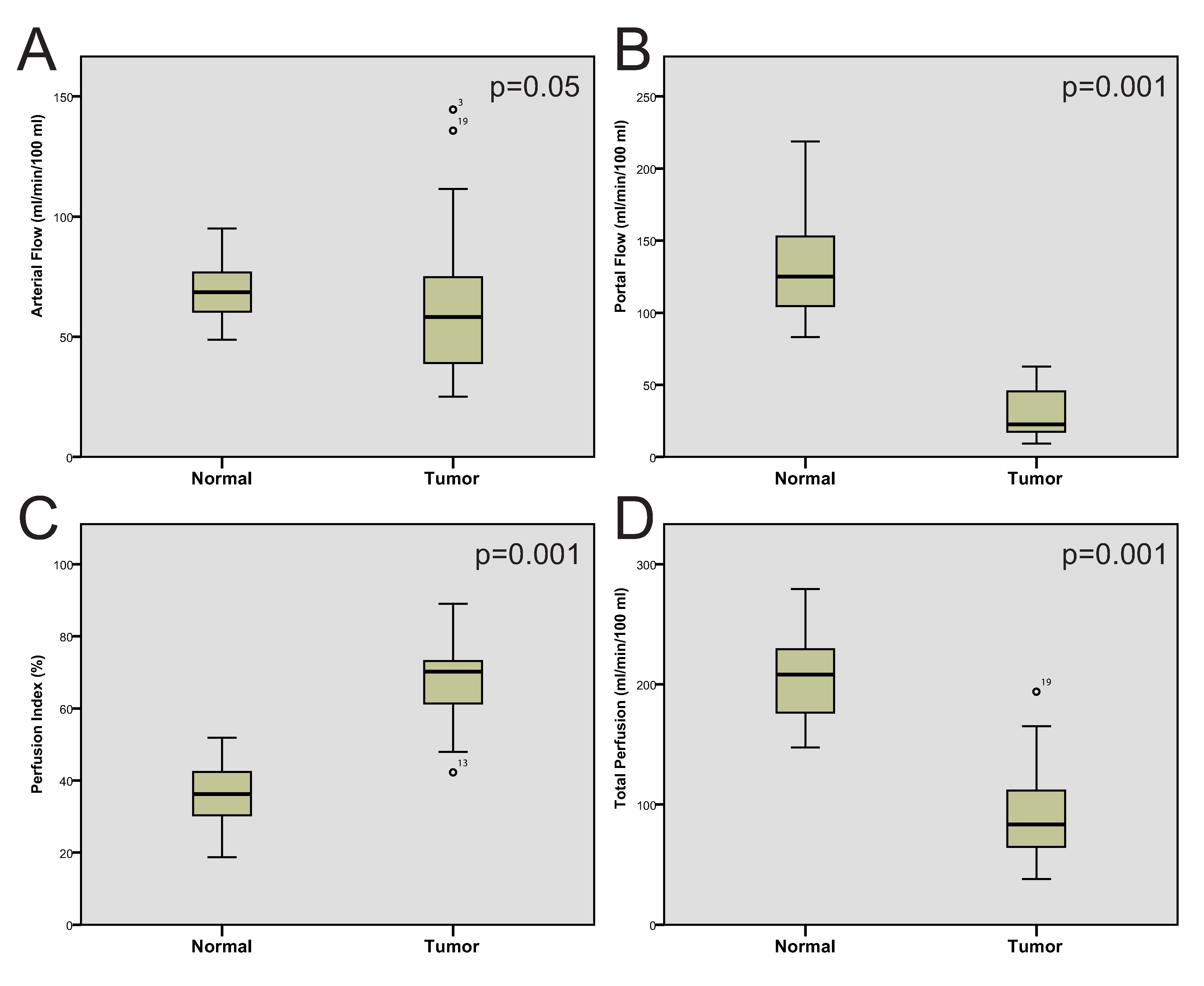

Supplement: S1 Fig — Box plots showing difference in arterial flow (A), portal flow (B), perfusion index (C) and the total perfusion (D) between tumor and normal hepatic tissue of studied patients. No significant difference was shown in arterial flow. Perfusion values of the tumor showed significantly lower portal flow, higher perfusion index and lower total perfusion compared to normal liver tissue. (TIF) [file pone.0245764.s001.tif]
